# Analysis of Reliability of Strain Measurements Made with the Fiber Bragg Grating Sensor Rosettes Embedded in a Polymer Composite Material

**DOI:** 10.3390/s21155050

**Published:** 2021-07-26

**Authors:** Valerii Matveenko, Natalia Kosheleva, Grigorii Serovaev, Andrey Fedorov

**Affiliations:** Institute of Continuous Media Mechanics, Ural Branch of Russian Academy of Sciences, 614068 Perm, Russia; mvp@icmm.ru (V.M.); serovaev@icmm.ru (G.S.); fedorov@icmm.ru (A.F.)

**Keywords:** strain rosette, fiber Bragg grating, strain, polymer composite material, numerical simulation

## Abstract

The results of strain measuring experiments, with the help of rosettes consisting of fiber Bragg grating sensors (FBG) embedded at the manufacturing stage in a polymer composite material are considered in this paper. The samples were made by the direct pressing method from fiberglass prepregs. A cross-shaped sample was tested under loading conditions corresponding to a complex stress state. A variant of strain calculations based on experimental data is discussed. The calculations were performed under the assumption of a uniaxial stress state in an optical fiber embedded in the material. The obtained results provide a reasonable explanation of the absence in the conducted experiment of two peaks in the reflected optical spectrum, the presence of which follows from the known theoretical principles. The experimental result with two peaks in the reflected optical spectrum was obtained for the same sample under a different loading scheme. The proposed variant of the numerical model of the experiment and the results of numerical simulation made for FBG rosettes embedded in the material allowed to estimate error in the strain values calculated on the assumption of the uniaxial stress state in the optical fiber and in the presence of two peaks in the reflected optical spectrum.

## 1. Introduction

Fiber-optic strain sensors (FOSS) based on fiber Bragg gratings (FBGs) are currently considered one of the most promising sensitive elements for strain measurement [1]. These elements, in comparison with other sensors, are more compact, do not require a separate power supply, and allow the inscription of several sensors in one fiber. The lack of sensitivity to electromagnetic influences allows using fiber-optic sensors in conditions where other types of sensitive elements are inapplicable [2]. The distinctive design features of fiber-optic sensors and their small size make it possible to embed the sensors into materials, the manufacturing technologies of which are associated with the transition from liquid to solid phase, and into materials manufactured by additive or powder technologies. The main limitation for embedding such sensor into the material is the temperature of the technological process, which should not lead to the loss of the operability of the fiber-optic sensor. Polymer composite materials (PCM) are, primarily, among such materials. In view of the variety of factors that determine the mechanical behavior of PCM, the embedding of FOSS into the material opens new possibilities for monitoring the state of a structure made from these materials, both at the manufacturing and operation stages.

The general solution to the problem of strain measurement by various sensors, including fiber-optic sensors, is associated with obtaining information about all components of the strain tensor. One way to solve this problem is to use special sensor layouts called rosettes [3,4,5,6,7]. When strain measurement is carried out inside or on the surface of a structure, being in the plane stress or plane strain state, the relevant information about all components of the strain tensor can be obtained using a rosette of three differently-directed sensors located on one plane. Fiber-optic rosettes can be created using one or more optical fibers. Construction of a rosette from one fiber implies the need to ensure the bending radii of the optical fiber, which should not lead to loss of the optical signal or destruction of the fiber. For rosettes, it is necessary to take into account that strain gradients in the sensor location area can lead to an error in the measurement result obtained on the basis of the rosette sensor readings. Different rosette schemes made from one optical fiber with FBGs are given in the patent [8].

Various works are devoted to the description of the strain measurement by rosettes made from Bragg grating sensors. One of the first is the work by [9], which presents the results of strain measurements by rosettes consisting of Bragg grating sensors located on a single optical fiber. The rosette was glued to the surface of the plate with epoxy adhesive. The strain in the plate was measured under different load levels on a four-point bending test machine. The significant number of studies is devoted to the description of rosette applications for measuring strains in specific products. For example, in work [10], rosettes were used to register strain in the shell frame of a power engine in complex dynamic regimes. It was shown that the FBG rosette, under severe operating conditions, is an effective substitute for the rosettes incorporating standard electrical strain gauges. The work by [11] demonstrates the use of FBG rosettes for strain monitoring of structural elements of ships exposed to wind, waves, and other loads. The rosette parameters were optimized based on the results of numerical simulation by the finite element method. The obtained strain values were compared with the results of measurements based on electrical resistance sensors. The comparison demonstrates good sensitivity and long-term stability of the proposed rosette design, which allows its application for monitoring the frame construction under corrosive and fatigue loading conditions. Applied problems associated with the use of FBG strain sensors as an alternative of electrical strain gauges are considered in works [12,13,14]. A variant of the weighting system with the FBG rosette designed for an object moving along the bridge is presented in [15]. Examples of the employment of FBG strain sensors for measuring strains in different products are given in works [16,17,18,19]. It is noted that maximizing the angle between the axes of the sensors increases the accuracy of the obtained information on strains, and a decrease in the bending radius of the fiber leads to a decrease in the fatigue life of the fiber.

A number of works focus on studying the problems concerning the use of rosettes from FBG sensors and new possibilities of their practical application. In [20], FBG rosettes are used for strain monitoring on the surface of a structure and considered an alternative to the traditional sensors. The paper raises the problem of sensor mounting to the surface of the object, which, thus far, has not been given due attention and establishes the dependence of the strain measurements on the mechanical characteristics of the glue, which are additionally evaluated using the method of digital image correlation. In [21], three Bragg grating sensors are used to determine the direction of reflected acoustic waves. Of particular interest are the works dealing with FBG rosettes embedded into the material. The authors of [22] describe an intelligent plate made of a composite material with three FBG sensors, which were embedded in the plate at the manufacturing stage forming a rosette with a 45 degree angle between sensors. On the basis of such plates, installed in the ship’s hull at the sites of application of high loads, a sensor network is created, which provides prediction of structural failure. In [23], an FBG rosette embedded into the material provides strain measurement in samples during the process of drilling holes. The obtained information underlay the experimental method of residual stress analysis, which was based on the hole drilling method.

A number of works discuss technologies for creating sensor networks, including those with the implementation of FBG-based rosettes. An example of such studies is in work [24], which presents the results of experiments with an FBG array orthogonally embedded in composite structures. In [25], the use of an FBG array in the form of rosettes embedded in composite sandwich panels is presented. The obtained results of measuring strains under impact loads were used in the design of the FBG array embedded into the hull of a high-speed boat. Worthy of notice are the results of work [26], where the authors study the influence of negative temperatures and relative humidity on FBG sensors, used in the configuration of rosettes embedded in glass fiber reinforced composite.

One of the key problems associated with application of FBGs embedded in the material is the evaluation of strains based on information about the registered physical quantity. The known relations establish the direct relationship between the measured strains and the readings of sensors only in the case of a uniaxial stress state in the location of the Bragg grating. In review paper [27], which extensively focuses on this problem, the authors note that, in the presence of FBGs embedded in the material, the strain fields do not correspond to the uniaxial stress state, and the results of the strain calculation obtained in this case must be calibrated. As a constructive solution for obtaining reliable information about the three components of the strain tensor in an optical fiber, the option of using a pair of micro-structured FBGs is considered in [28,29]. One of the options for establishing the relationship between strains in a composite material and in an optical fiber is considered in [30]. The authors proposed introducing a transfer matrix on the basis of numerical simulation, the components of which are calculated based on information on three independent tests and establish the relationship between strains in the material and optical fiber.

The authors of [31] describe the results of mathematical modeling, which makes it possible to estimate the error limits of stain calculation based on the assumption of a uniaxial stress state in an FBG embedded into the material. An additional problem for FBG sensors embedded into the material and using the assumption of a uniaxial stress state in the Bragg grating zone to calculate strains, is the possibility of appearance of two peaks in the reflected spectrum of the optical signal, which is the result of the birefringence effect. Examples of theoretical and experimental studies related to this effect are works [32,33,34].

In the present work, experimental results on the strain measurement by rosettes made from FBG sensors embedded in a polymer composite sample under a complex stress state are presented. The schemes and results of numerical simulation that interpret the experiment are considered. A variant of estimating error limits of strain, obtained using assumptions that allow calculating the strain values on the basis of measured physical quantities, is considered.

## 2. Strain Measurement with Fiber Bragg Grating Rosettes Embedded in a Polymer Composite Sample under Biaxial Tension

**Figure 1:**
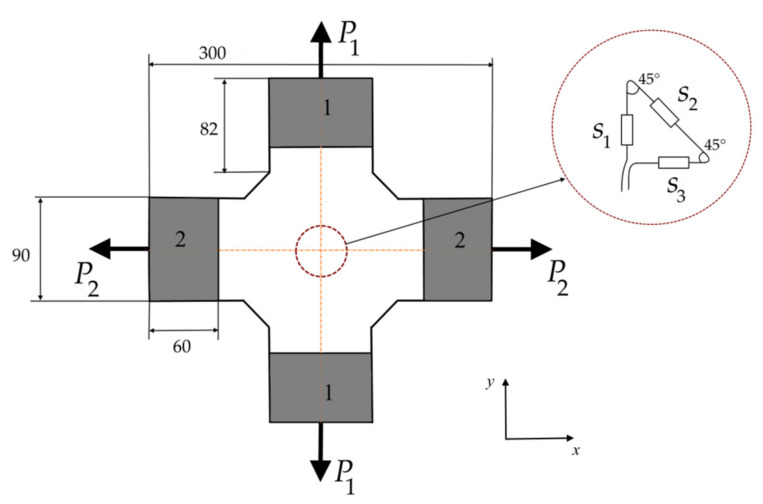
Experimental cross-shaped sample with embedded rosette consisting of sensors $s_{1},\;s_{2},\;s_{3}$, located at the single optical fiber.

A cross-shaped PCM sample was used in the experiment on strain measurements with the embedded fiber Bragg grating sensors arranged in a rosette configuration (Figure 1). A complex stress state is achieved in such samples by testing on biaxial tensile machines. To obtain reliable strain values, it is necessary to exclude large strain gradients from the rosette region. The results of numerical simulation by the finite element method of the stress–strain state in the sample at different combinations of loads $P_{1}$ and $P_{2}$ allow one to conclude that for the prescribed dimensions of the sample a uniform stress state is established in the zone of rosette location. Experiments were carried out for three samples. Each sample contained an embedded single optical fiber with three FBGs laid out in the form of rosettes, according to the scheme in Figure 1. Samples were made from one batch of material. The embedded FOSS had the same parameters for each of the samples. The difference in the results was within 5%.

The selected shape of the sample, under the condition of elastic material behavior in the range of the tested loads, makes it possible to obtain experimental results for the complex stress state when tested on uniaxial tensile machines. This case was used in the present work. The equivalent of biaxial tension by forces $P_{1}$ and $P_{2}$ would be the sum of two experiments: loading with forces $P_{1}$ by gripping zones 1 and loading with forces $P_{2}$ by gripping zones 2.

PCM samples were manufactured by direct pressing method from 20 layers of fiberglass prepreg with the following mechanical properties: tensile modulus of elasticity along the warp direction $E_{xx}=23.5\;\mathrm{GPa}$; tensile elasticity modulus in weft direction $E_{yy}=23.5\;\mathrm{GPa}$; tensile elasticity modulus perpendicular to the layer plane $E_{zz}=6\;\mathrm{GPa};$ shear moduli: $G_{xy}=3\;\mathrm{GPa},$ $G_{xz}=3\;\mathrm{GPa},$ $G_{yz}=3\;\mathrm{GPa};$ Poisson’s ratios: $\nu_{12}=0.14$, $\nu_{13}=0.13$, $\nu_{23}=0.13$. The sample with 20 layers of prepreg has a thickness of 5 mm.

A rosette consisting of three fiber Bragg grating sensors was embedded into the PCM during the technological process of material formation at the stage of stacking the prepreg layers on the molding tool. The sensor rosette was placed between the second and third layers of prepreg. The single-mode bend insensitive germanosilicate optical fibers of the SM1500(9/125)P series with photosensitivity were used. Silica glass optical fiber has the following mechanical properties: elastic modulus $E_{f}=71.4\;\mathrm{GPa}$, Poisson’s ratio $\nu_{f}=0.17$. It has a diameter of 0.125 ± 0.002 mm and a protective coating with a thickness of 0.010 ± 0.0025 mm, which is made of polyimide with mechanical properties $E_{c}=2.7\;\mathrm{GPa}$, $\nu_{c}=0.31$. Fiber Bragg gratings were inscribed by means of ultraviolet source by the phase mask method [35], followed by overcoating the grating area with a polyimide material. The used sensors have a Bragg grating length of 5 mm. For these sensors, the selected rosette dimensions ensure the minimum size of the strain measurement zone, and that the bending radius does not distort the optical signal transmitted through the fiber.

The main property of a Bragg grating consists in reflecting the part of a broadband optical signal transmitted through an optical fiber. The value of strains measured with the FBG sensors is obtained by processing information about the resonant wavelength of the reflected signal recorded by the interrogator. The resonant wavelength of the reflected signal is determined by the effective refractive index n of the optical fiber core in the zone of the grating and the period of the grating structure Λ:

(1) $$\lambda^{*}=2n\Lambda .$$

The interaction of the fiber-optic sensor with a deformable material causes the length of the Bragg grating to change, leading to a change in the resonant wavelength of the reflected signal. The relationship between the change in the resonant wavelength of the reflected spectrum and the strain of the fiber in the Bragg grating zone is determined by the relations [27]:

(2) $$\begin{array}{l} \frac{\Delta \lambda_{1}}{\lambda_{*}}=\varepsilon_{3}-\frac{1}{2}n_{}^{2}(p_{11}\varepsilon_{1}+p_{12}(\varepsilon_{2}+\varepsilon_{3}))\;,\\ \frac{\Delta \lambda_{2}}{\lambda_{*}}=\varepsilon_{3}-\frac{1}{2}n_{}^{2}(p_{11}\varepsilon_{2}+p_{12}(\varepsilon_{1}+\varepsilon_{3}))\;, \end{array}$$

where $\varepsilon_{3}$ is the strain along the fiber, $\varepsilon_{1},\varepsilon_{2}$ are the principal strains in the plane perpendicular to the optical fiber. $\Delta \lambda_{1}=\lambda_{1}-\lambda_{*},$ $\Delta \lambda_{2}=\lambda_{2}-\lambda_{*}$ are the difference in the resonant wavelengths of the reflected spectrum at the current ($\lambda_{1},\lambda_{2}$) and initial ($\lambda_{*}$) moments of time (the initial and current moments of time during measurements correspond to the undeformed and deformed states), $p_{11},\;p_{12}$ are strain-optic coefficients. For the FOSS used in the experiment, n=1.458, $p_{11}=0.121$, $p_{12}=0.270$ [34].

In the uniaxial stress state, the strains in the optical fiber, which does not interact with the environment, are, $\varepsilon_{1}=\varepsilon_{2}=-\nu \varepsilon_{3}$, where ν is the Poisson’s ratio of the optical fiber. In this case $\Delta \lambda_{1}=\Delta \lambda_{2}=\Delta \lambda$ and

(3) $$\frac{\Delta \lambda }{\lambda_{*}}=\left(1-\frac{n^{2}}{2}\left(p_{12}-\nu (p_{11}+p_{12})\right)\right)\varepsilon_{3},$$

or

(4) $$\varepsilon_{3}=\frac{1}{k}\cdot \frac{\Delta \lambda }{\lambda_{*}}.$$

For used optical fibers, k=0.78.

Relations (2) and (4) show that an unambiguous relationship between the experimental data on the change of the resonant wavelength of the reflected signal, and the component of the strain tensor in the fiber along its length, takes place only in the case of a uniaxial stress state in the region of the Bragg grating. In the general case, a complex stress state with three different components of the strain tensor: $\varepsilon_{1},\;\varepsilon_{2},\;\varepsilon_{3}$ is established in the optical fiber embedded into the material. In this case, it is impossible to determine the value of the strain $\varepsilon_{3}$ using relations (2). One of the most commonly encountered approaches for obtaining the values of measured strains is based on the use of relation (4). The above condition is satisfied when using a design of sensors, in which a section of the optical fiber with a Bragg grating is placed in a capillary to keep this section in the uniaxial stress state [32]. Despite the obvious simplicity and advantages of this option, there are a few disadvantages that should be mentioned. The presence of a capillary increases the size of the sensor structure, which leads to a significant increase in the stress concentration in the vicinity of the sensor embedded into the material, which might have negative effect on a PCM [36]. In addition, the use of the capillary gives rise to the problem of meeting the main requirement for a sensor design, namely, the strains along the fiber in the Bragg grating and in the adjacent material must coincide. When an optical fiber is embedded in a material, the zone of the Bragg grating stays in contact with the material, and the condition of a uniaxial stress state in the Bragg grating is taken as an assumption. In this case, the error introduced by this assumption is estimated and eliminated by different methods, in particular, by establishing calibration coefficients. In this work, the variant of using relation (4) to obtain the values of the measured strain at ideal contact of the optical fiber with the material is used, analyzed, and evaluated.

Relation (2) shows that for fiber Bragg gratings embedded into the material in a complex stress state, there will be two resonant wavelengths of the reflected spectrum $\lambda_{1}$ and $\lambda_{2}$, which should lead to the occurrence of two peaks in the reflected spectrum, which is recorded by the interrogator. In this case, the accepted assumption of a uniaxial stress state in the zone of the Bragg grating poses the problem of choosing a value of $\Delta \lambda /\lambda_{*}$, which determines the value of the measured strain $\varepsilon_{3}$ in expression (4).

**Figure 2:**
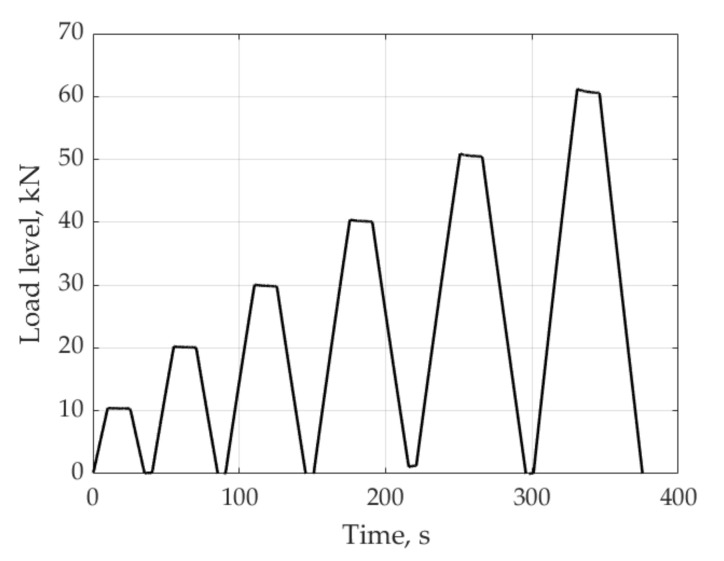
Scheme of sample loading.

**Figure 3:**
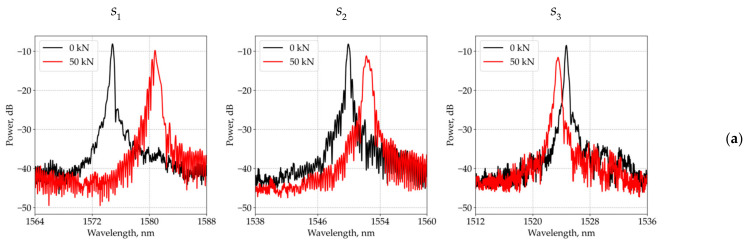

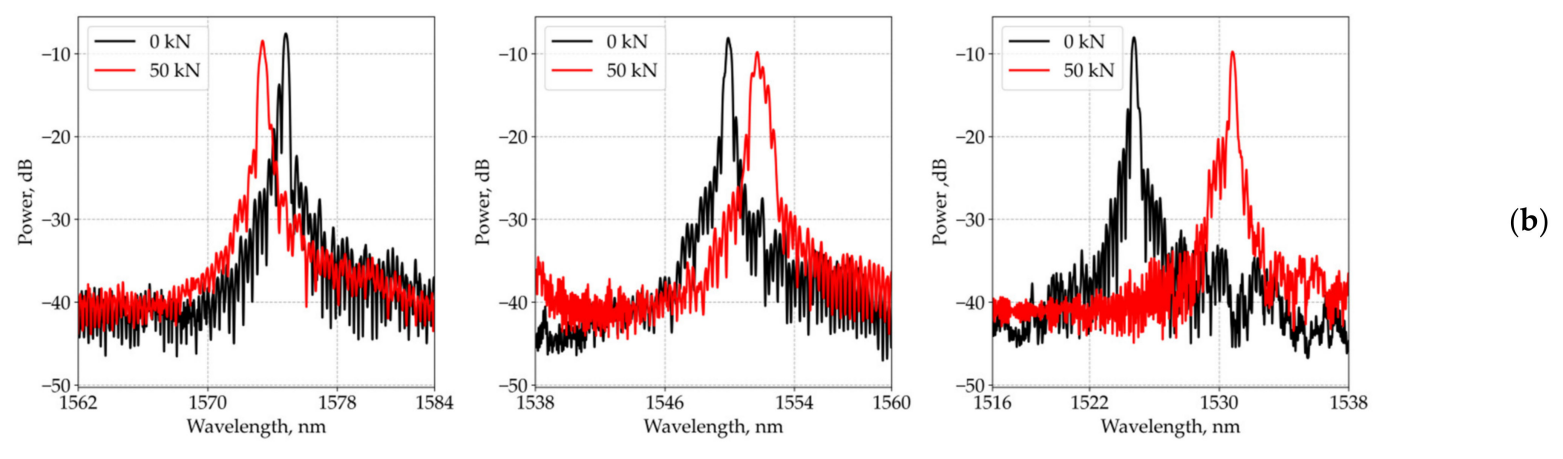
Reflected optical spectra in the unloaded state and under load $P_{0}=50\;\mathrm{kN}$ in direction 1 (**a**) and direction 2 (**b**).

The tension is applied to the studied sample in direction 1 by gripping zones 1 and in direction 2 by gripping zones 2 (Figure 1). The loading diagram is shown in Figure 2. At all load levels in directions 1 and 2, despite some distortion, the above mentioned distinct peaks are not observed in the reflected spectrum. As an example, Figure 3 shows the reflected optical spectra for the first ($s_{1}$), second ($s_{2}$), and third ($s_{3}$) sensors in the unloaded state and under load in directions 1 and 2. At loads over 60 kN, the first signs of material failure appear. Up to this loading level, the patterns of the reflected spectra are similar. For their demonstration, in Figure 3, a variant under a load equal to 50 kN is shown.

**Figure 4:**
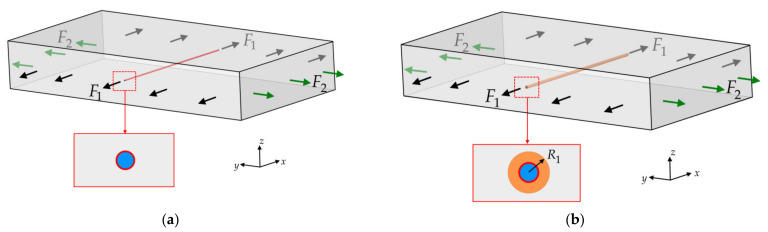
PCM models with embedded optical fiber: without resin pocket (**a**) and with resin pocket (**b**).

The absence of two peaks in the reflected spectrum can be explained by the results of the following numerical experiments. Consider a PCM element shown in Figure 4a, which completely reflects the distribution of the stress–strain state in the sample, where the FOSS is embedded. An optical fiber with a Bragg grating is embedded into the element, which is in perfect contact with the material. The mechanical behavior of the PCM is described by the model of an elastic orthotropic body with a tensile modulus along the *x*, *y*, *z* axes $E_{xx}=E_{yy}=23.5\;\mathrm{GPa}$, $E_{zz}=6\;\mathrm{GPa},$ Poisson’s ratios $\nu_{xy}=0.14$, $\nu_{yz}=\nu_{xz}=0.13,$ shear moduli $G_{xy}=G_{yz}=G_{xz}=3\;\mathrm{GPa}.$ Based on the finite element method in the framework of the ANSYS engineering package for a segment with an embedded optical fiber, the stress–strain state was calculated under loads acting along the optical fiber ($F_{1}=P_{0}$, $F_{2}=0$) and perpendicular to the optical fiber ($F_{1}=0$, $F_{2}=P_{0}$). The values of strains $\varepsilon_{1},\;\varepsilon_{2},\;\varepsilon_{3}$, which are calculated in the zone of the Bragg grating according to relations (2), are used to find theoretical values of $\Delta \lambda_{1}/\lambda_{*}$, $\Delta \lambda_{2}/\lambda_{*}$. Table 1 shows the results obtained for $P_{0}$ corresponding to loads $P_{1}$ and $P_{2}$ of 50 kN.

The presented results demonstrate that under loads acting along the optical fiber, in contrast to the load acting perpendicular to the fiber, a stress state close to uniaxial takes place in the zone of the Bragg grating. In this case, relation (2) shows that $\Delta \lambda_{1}/\lambda_{*}\approx \Delta \lambda_{2}/\lambda_{*}$, which corresponds to one peak in the reflected spectrum.

**Figure 5:**
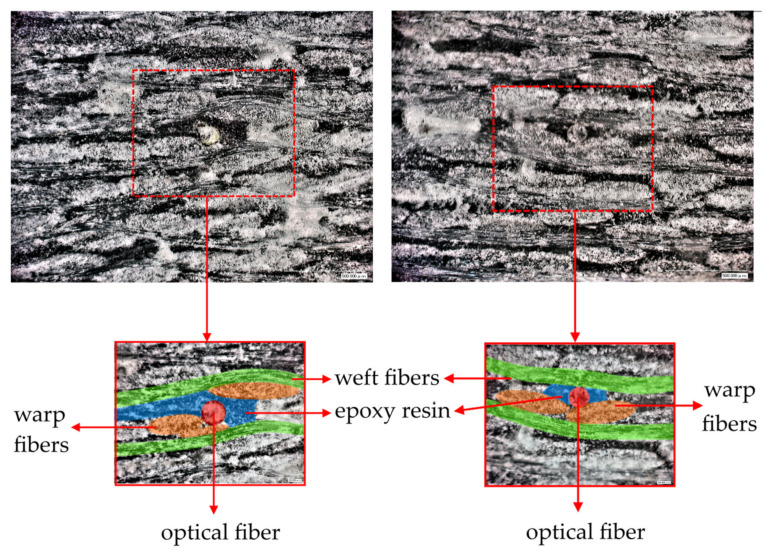
Photos of sample cross-sections.

Known theoretical and experimental results have shown that embedding of an optical fiber in a PCM can produce a technological defect called a resin pocket [37]. This defect is the formation of a matrix-filled area around the fiber. Mechanical characteristics of the matrix: $E_{m}=2\;\mathrm{GPa}$, $\nu_{m}=0.35$. The geometry of this region depends on the PCM reinforcement scheme and fiber orientation relative to the reinforcement direction. To assess the structure of the material in the vicinity of the optical fiber embedded into the PCM, microscopic images of the cross sections of the samples perpendicular to the optical fiber were obtained. Figure 5 shows cross-sectional images at 140× magnification. These results allow one to conclude that there is a region around the optical fiber filled with matrix and comparable in size to the optical fiber. This result became the basis for correcting the PCM model shown in Figure 4a with embedded optical fiber. Figure 4b shows a variant of the model, which has a region around the fiber filled with epoxy resin. The geometry of this region is determined by the value $\alpha =R_{1}/R_{0}$, where $R_{0}$ is the radius of the optical fiber, $R_{1}$ is the outer radius of the region filled with matrix.

**Figure 6:**
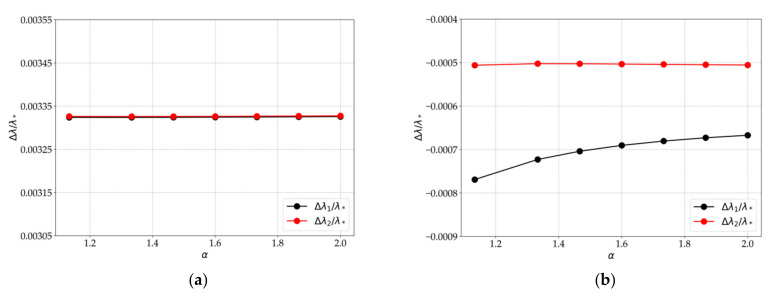
The values of $\Delta \lambda_{1}/\lambda_{*}$ and $\Delta \lambda_{2}/\lambda_{*}$ at different values of α and loads $F_{1}=P_{0}$, $F_{2}=0$ (**a**); $F_{1}=0$, $F_{2}=P_{0}$ (**b**).

For the refined PCM model with an embedded optical fiber and a region filled with matrix, the strains in the Bragg grating zone were calculated and the corresponding theoretical values of $\Delta \lambda_{1}/\lambda_{*}$, $\Delta \lambda_{2}/\lambda_{*}$ were obtained at different values of α, which are shown in Figure 6.

The obtained results show that in the case of applying loads along the optical fiber, $\Delta \lambda_{1}/\lambda_{*}$ and $\Delta \lambda_{2}/\lambda_{*}$ are practically equivalent for different values of α, and at loads applied perpendicular to the optical fiber, the reflected spectrum has two peaks that approach each other with an increase in the matrix layer size. In addition, a quantitative analysis of the values $\Delta \lambda_{1}$ and $\Delta \lambda_{2}$, obtained for the mechanical characteristics of the material, optical fiber, and resin pocket shows that the value $\left|\Delta \lambda_{2}-\Delta \lambda_{1}\right|$ for different variants of loads referred to the experimental value $\lambda_{*}$ (Figure 3) is less than the width of the peak of the reflected spectrum. These results allow one to conclude that, in the implemented experiments, two peaks merge together in the total picture of the reflected spectrum.

**Figure 7:**
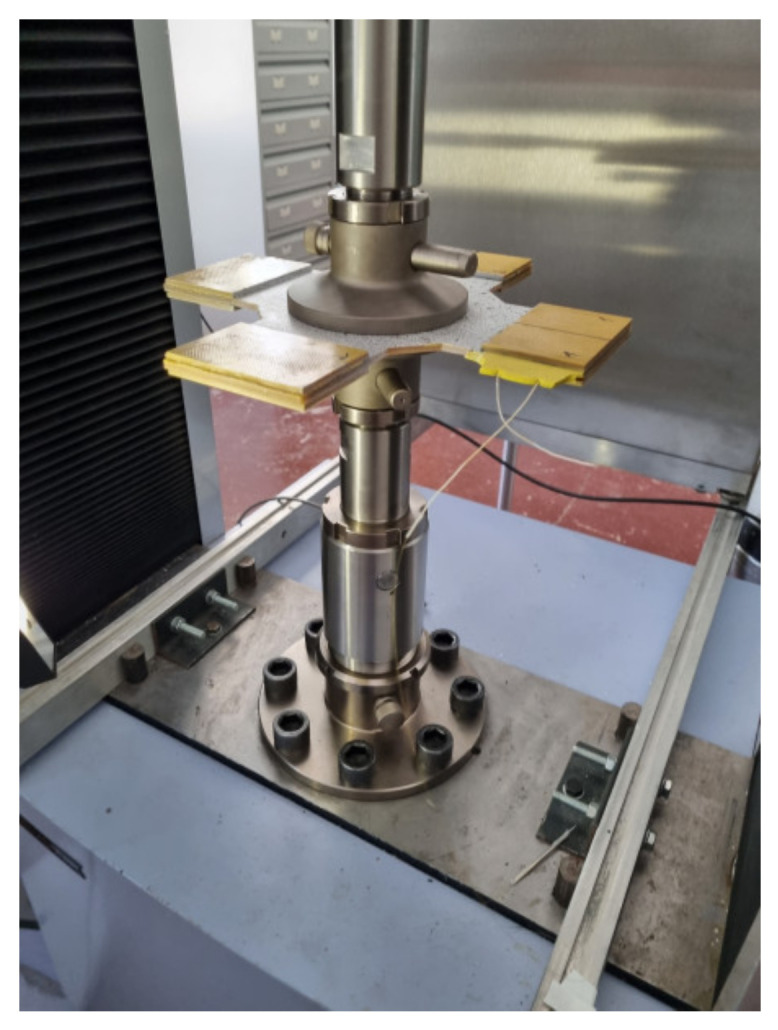
The scheme of loading.

**Figure 8:**
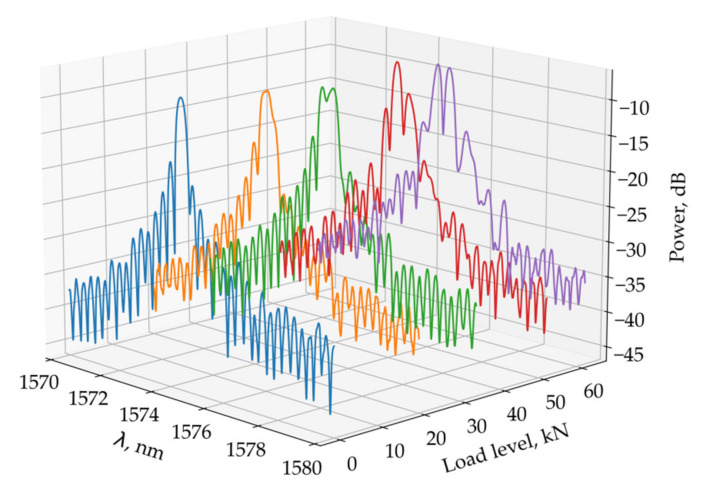
Reflected optical spectra of FBG under different loads.

The difference between the theoretical values $\Delta \lambda_{1}/\lambda_{*}$,$\Delta \lambda_{2}/\lambda_{*}$ increases with increasing load. For the selected loading schemes under loads that ensure the elastic behavior of the PCM, it was not possible to obtain two peaks in the reflected spectrum. For another loading scheme, when the load is distributed over the specimen surface (Figure 7), at a load level on the sample surface of more than 34 kN, the reflected spectrum shows two peaks at a distance that increases with increasing load. These results are shown in Figure 8.

**Figure 9:**
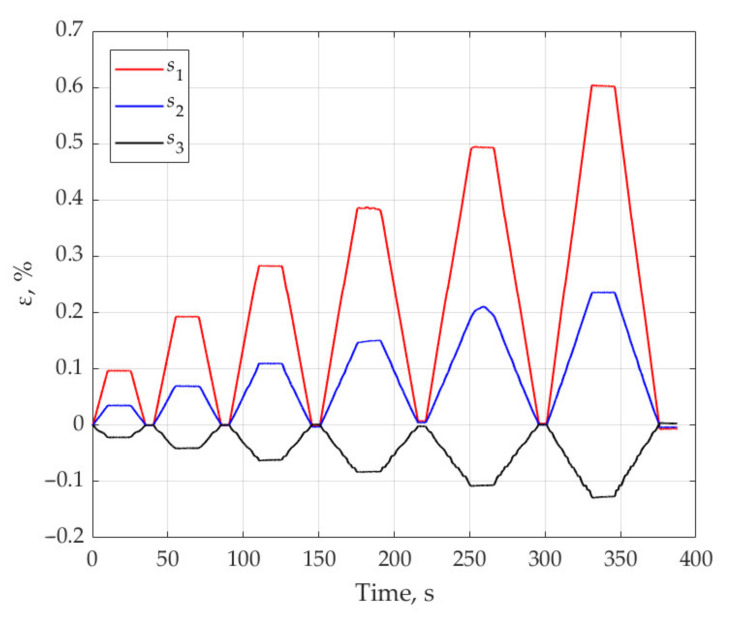
The strain values measured by the sensors $s_{1},\;s_{2},\;s_{3}$ when the sample was under tension in direction 1.

To obtain the strain values based on relation (4) for the sample subjected to tensile loading in directions 1 and 2, the maximum values of the resonant wavelengths in the reflected spectrum were used. For the loading scheme shown in Figure 2, Figure 9 shows the strain values measured by sensors $s_{1},\;s_{2},\;s_{3}$ when the sample is loaded in direction 1. These results demonstrate linear elastic behavior of the material at given load levels. In the experiments, in parallel with FBG sensors, the strains were measured with the VIC-3D optical system. The difference between the measurement results by the two methods did not exceed 6%.

**Figure 10:**
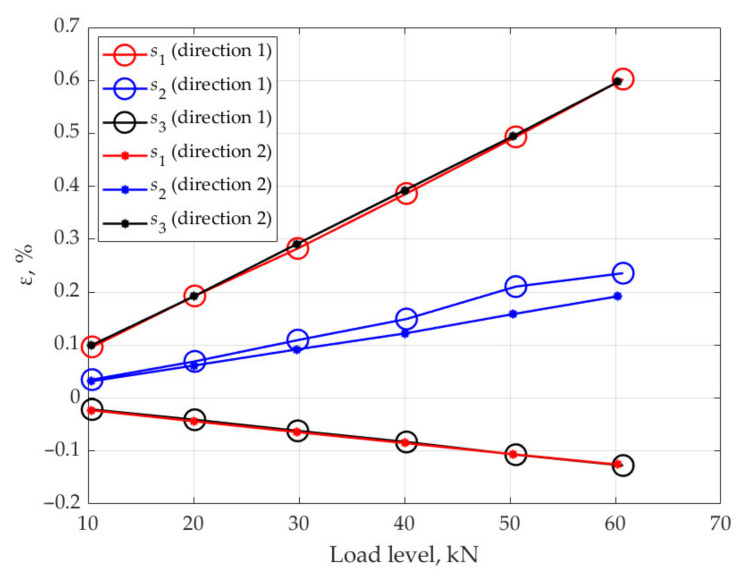
Strains at different values of loads in directions 1 and 2.

Figure 10 shows the strain values at different load levels under tension in the directions 1 and 2.

For the sample under consideration, and equivalent loads applied in directions 1 and 2, the readings of sensors $s_{1},\;s_{3}$ at loads applied along the direction 1 must coincide with the readings of sensors $s_{3}$ and $s_{1}$ at loads applied along the direction 2, and the readings of sensor $s_{2}$ must coincide at loads applied along directions 1 and 2. Analysis of the obtained results shows that the difference in the strain values obtained on the basis of the readings of sensors $s_{1}$ and $s_{3}$ under loading along directions 1 and 2 is within 6%, and the strains obtained on the basis of readings of sensor $s_{2}$ under loading along directions 1 and 2 differ by 25%. A considerable difference in strains obtained from the readings of the sensor $s_{2}$ can be explained by the fact that the orientation angle of the sensor $s_{2}$ relative to the loading axis differs from 45°. This assumption follows from the analysis of the equations for the rosettes:

(5) $$\begin{array}{l} \varepsilon_{3}^{1}=\varepsilon_{x}\cos^{2}\theta_{1}+\varepsilon_{y}\sin^{2}\theta_{1}+\gamma_{xy}\sin\theta_{1}\cos\theta_{1},\\ \varepsilon_{3}^{2}=\varepsilon_{x}\cos^{2}\theta_{2}+\varepsilon_{y}\sin^{2}\theta_{2}+\gamma_{xy}\sin\theta_{2}\cos\theta_{2},\\ \varepsilon_{3}^{3}=\varepsilon_{x}\cos^{2}\theta_{3}+\varepsilon_{y}\sin^{2}\theta_{3}+\gamma_{xy}\sin\theta_{3}\cos\theta_{3}, \end{array}$$

where $\theta_{1},\;\theta_{2},\;\theta_{3}$ are the angles between the x-axis and the orientation directions of the sensors $s_{1},\;s_{2},\;s_{3}$; $\varepsilon_{3}^{1},\;\varepsilon_{3}^{2},\;\varepsilon_{3}^{3}$ are the strains along the optical fiber for sensors $s_{1},\;s_{2},\;s_{3}$ respectively.

The obtained close values for strains $\varepsilon_{3}^{1}$ and $\varepsilon_{3}^{3}$ under loading along directions 1 and 2 suggest that the values of the angle $\theta_{1}=90{}^{\circ}$ and $\theta_{3}=0$ are provided when the rosette is embedded in the material. Taking into account that $\varepsilon_{3}^{1}=\varepsilon_{y}$, $\varepsilon_{3}^{3}=\varepsilon_{x}$ from Equation (5), the relation for the angle $\theta_{2}$

$$\cos^{2}\theta_{2}=\frac{\varepsilon_{3}^{2}-\varepsilon_{3}^{1}}{\varepsilon_{3}^{3}-\varepsilon_{3}^{1}}.$$

This relation shows that the value of the angle $\theta_{2}$, based on the values of the strains under loading along direction 1, is 43°, and under loading along direction 2, is 48°. These results indicate that the angle $\theta_{2}$ is different from 45°.

These results demonstrate that when using rosettes embedded into the material, small errors in the information about the orientation angle of the sensors lead to an additional error in the determined strains.

## 3. Results of a Numerical Model of the Experiment on Measuring Strains by Fiber Bragg Grating Rosettes Embedded into the Material

Relation (2), which determines the relationship between the strain tensor components in a fiber Bragg grating and the characteristics of the reflected spectrum, generally demonstrate the presence of two peaks in the spectrum. In the case of a uniaxial stress state in an optical fiber, relation (4) determines an unambiguous relation between the strain in the optical fiber and one resonant wavelength in the reflected optical spectrum. The assumption of a uniaxial stress state, which is used for strain calculations in an embedded optical fiber and the presence of two peaks in the reflected spectrum may cause a problem with their choice for relation (4).

**Figure 11:**
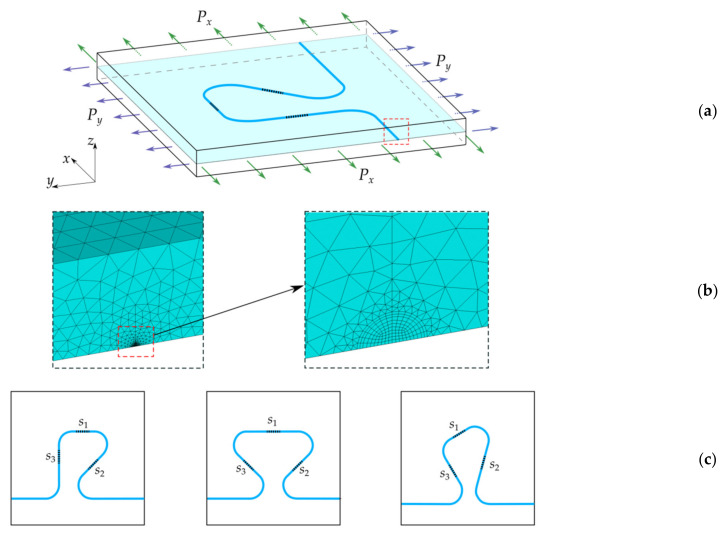
Calculation schemes for PCM with an embedded optical fiber forming a rosette of sensors (**a**), examples of discretization of a finite element mesh of the region near the optical fiber (**b**), options for sensor location in the rosette (**c**).

Numerical experiments can provide additional information on the error limits, in the case of using in relation (4) the information about each of the peaks in the reflected optical spectrum. A plate with an embedded rosette (Figure 11a) is considered for the numerical modeling under different types of loading applied to the side faces. The numerical model does not take into account the presence of the resin pocket. In accordance with the previously presented results, the disregard of the resin pocket leads to an upper error limit for the calculated strains. In simulation, the material is represented as a homogeneous medium with effective mechanical characteristics of the considered PCM. The plate thickness is 5 mm, which corresponds to the experimental sample. The planar dimensions of the model define the zone of the uniform stress state in Figure 1. The geometric and mechanical characteristics of the optical fiber are given in the previous section. Figure 11c shows the variants of rosette configurations, in which sensors have different orientations relative to external loads considered in the numerical simulation.

The results of the numerical experiments are the values of the characteristics of the reflected optical spectrum $\Delta \lambda_{1}^{i}/\lambda_{*}^{i}$ and $\Delta \lambda_{2}^{i}/\lambda_{*}^{i}$ (i=1,2,3 corresponds to sensors $s_{1},\;s_{2},\;s_{3}$) calculated using relation (2) for the values of the strain tensor components $\varepsilon_{1}^{ij},\;\varepsilon_{2}^{ij},\;\varepsilon_{3}^{ij}$ (j=1,2 corresponds to resonant wavelength shift $\Delta \lambda_{1}^{i}$ and $\Delta \lambda_{2}^{i}$ respectively) in fiber Bragg gratings obtained from the calculation of the stress–strain state of a plate with an embedded optical fiber. The calculations were carried out by the finite element method in a three-d formulation using meshes that ensure the required accuracy of calculations in the optical fiber and its neighborhood. A fragment of the finite element discretization with the refinement of mesh elements to the fiber is shown in Figure 11b.

The values $\Delta \lambda_{1}^{i}/\lambda_{*}^{i}$ and $\Delta \lambda_{2}^{i}/\lambda_{*}^{i}$ obtained in the numerical experiment are used to calculate the strains measured by the sensors $s_{1},\;s_{2},\;s_{3}$ on the basis of relation (4). The result of these calculations, based on the values $\Delta \lambda_{1}^{i}/\lambda_{*}^{i}$ corresponding to the first peak in the reflected optical spectrum, will be strains $\varepsilon_{3}^{11}$, $\varepsilon_{3}^{21}$, $\varepsilon_{3}^{31}$ and based on the values $\Delta \lambda_{2}^{i}/\lambda_{*}^{i}$—strains $\varepsilon_{3}^{12}$, $\varepsilon_{3}^{22}$, $\varepsilon_{3}^{32}$. Finally, using the relation (5) for rosettes, the components of the strain tensor $\varepsilon_{x}^{1}$, $\varepsilon_{y}^{1}$, $\varepsilon_{xy}^{1}$ corresponding to $\Delta \lambda_{1}^{i}/\lambda_{*}^{i}$ and strains $\varepsilon_{x}^{2}$, $\varepsilon_{y}^{2}$, $\varepsilon_{xy}^{2}$ corresponding to $\Delta \lambda_{2}^{i}/\lambda_{*}^{i}$ are calculated.

Analysis of the results of numerical experiments showed the fulfillment of one of the main properties of the rosettes, namely, the strain values $\varepsilon_{x}^{1}$, $\varepsilon_{y}^{1}$, $\varepsilon_{xy}^{1}$ and $\varepsilon_{x}^{2}$, $\varepsilon_{y}^{2}$, $\varepsilon_{xy}^{2}$ coincide for all versions of the rosettes geometries shown in Figure 11c. This result is one of the demonstrations of reliability of the performed numerical simulation.

Comparison of the strain values $\varepsilon_{x}^{1}$, $\varepsilon_{y}^{1}$, $\varepsilon_{x}^{2}$, $\varepsilon_{y}^{2}$, obtained in a numerical experiment with material strains $\varepsilon_{x}^{}$, $\varepsilon_{y}^{}$ in the zone of rosette’s location allows to estimate the error in the strain calculation, which is the result of using the assumption of a uniaxial stress state in the area of FBG rosette. As a measure of these errors, the following quantities were introduced:

(6) $$\delta_{x}^{1}=\frac{\varepsilon_{x}^{1}-\varepsilon_{x}}{\varepsilon_{0}}\cdot 100\%,\;\delta_{y}^{1}=\frac{\varepsilon_{y}^{1}-\varepsilon_{y}}{\varepsilon_{0}}\cdot 100\%\;\delta_{x}^{2}=\frac{\varepsilon_{x}^{2}-\varepsilon_{x}}{\varepsilon_{0}}\cdot 100\%,\;\delta_{y}^{2}=\frac{\varepsilon_{y}^{2}-\varepsilon_{y}}{\varepsilon_{0}}\cdot 100\%$$

where $\varepsilon_{0}$ is the strain of the material in the rosette zone under uniaxial loading $P_{0}$.

**Figure 12:**
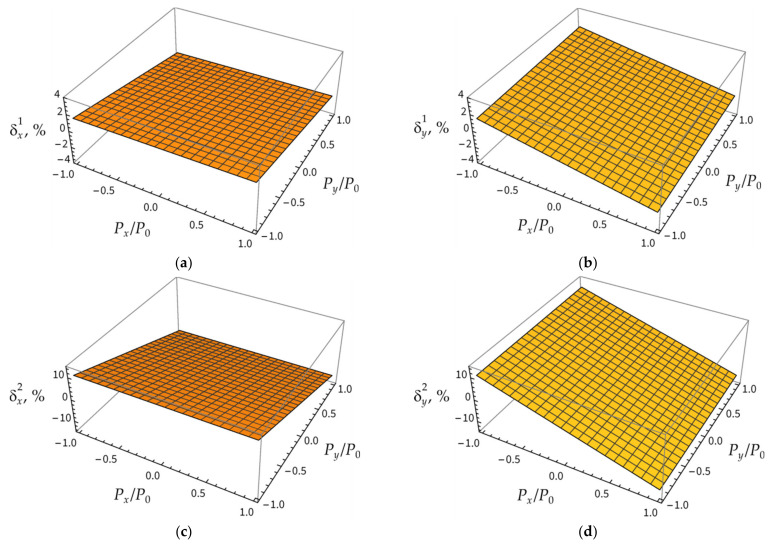
The values of errors $\delta_{x}^{1}$(**a**), $\delta_{y}^{1}$ (**b**), $\delta_{x}^{2}$ (**c**), $\delta_{y}^{2}$ (**d**) at different loads $P_{x}/P_{0}$ and $P_{y}/P_{0}$.

Figure 12 shows the values of errors (6) at different loads $P_{x}/P_{0}$ and $P_{y}/P_{0}$ on the side faces.

From the presented results, it can be seen that, according to (6), the error of the calculated values of strains is much less when $\Delta \lambda_{1}^{i}/\lambda_{*}^{i}$ is used in relation (4) rather than $\Delta \lambda_{2}^{i}/\lambda_{*}^{i}$. However, there are no methods for selecting a peak in the reflected spectrum that will provide the smallest error in the strain calculation by relation (4). Analysis of the obtained results suggests that, in the presence of two peaks in the reflected spectrum, it is reasonable to use, for relation (4), the arithmetic mean value of $\Delta \lambda_{1}^{i}/\lambda_{*}^{i}$ and $\Delta \lambda_{2}^{i}/\lambda_{*}^{i}$. In this case, for any load types, the error in strain values calculated by (6) does not exceed 6%.

## 4. Conclusions

Experimental and theoretical results on the strain measurement by FBG-based rosettes embedded into PCM are presented. The rosette was embedded into the material at the technological stage of its manufacturing by the method of direct pressing of fiberglass prepregs. A cross-shaped sample was loaded by forces applied in its plane in different directions. The strains were calculated based on the measurements of physical quantities of the reflected optical spectrum under the assumption of a uniaxial stress state in an optical fiber embedded in the material. The reliability of the presented results of strain measurements with FBG sensors is confirmed by satisfactory agreement with the results obtained by the VIC-3D optical system.

In contrast to the theoretical results predicting the existence of two peaks in the reflected optical spectrum, the spectra obtained in the experiments only have one peak. To clarify this contradiction, the results of numerical simulations are presented, which qualitatively and quantitatively explain the difference between the theoretical and experimentally obtained values of resonant wavelengths of the reflected optical spectra. On the considered sample under loads acting perpendicular to the plane of the sample, a variant with two peaks in the reflected optical spectrum was experimentally obtained. The analysis of the obtained experimental results using numerical simulation methods shows that the error in the information about the angles of orientation of the sensors has a significant effect on the results of strain calculations based on the relations for rosettes. It should be noted that the numerical simulation conducted under the assumption of a uniaxial stress state in an optical fiber embedded in the material and the existence of two peaks in the reflected optical spectrum poses the problem of selecting one of them to calculate the strains. A numerical model of an experimental sample with an embedded rosette was developed. The numerical simulation results of the error in the strain calculations under the assumption of a uniaxial stress state in an optical fiber, as well as information on the theoretical reflected spectrum with two peaks, were analyzed. The obtained results demonstrate that information about one of the peaks provides a significantly smaller error in calculating the strain values. However, there were no adequate methods for selecting a peak, securing a smaller error in the strain values. Therefore, in the case when the reflected spectrum has two peaks, it is suggested that the strain calculations are made using their arithmetic mean value.

## Figures and Tables

**Table 1:** The results of numerical calculations of strain values and resonant wavelength shift for PCM model with embedded optical fiber without resin pocket.

Load Scheme	$\varepsilon_{1}$	$\varepsilon_{2}$	$\varepsilon_{3}$	$\Delta \lambda_{1}/\lambda_{*}$	$\Delta \lambda_{2}/\lambda_{*}$	$\left|\Delta \lambda_{2}-\Delta \lambda_{1}\right|,\;\mathrm{nm}$
$F_{1}=P_{0}$, $F_{2}=0$	−0.0007	−0.0007	0.0043	0.003318	0.003320	0.0034
$F_{1}=0$, $F_{2}=P_{0}$	−0.0004	0.0015	−0.0006	−0.000824	−0.000519	0.4730

## Data Availability

Not applicable.
